# Fascin-1 is a novel biomarker of aggressiveness in some carcinomas

**DOI:** 10.1186/1741-7015-11-53

**Published:** 2013-02-26

**Authors:** Vathany Kulasingam, Eleftherios P Diamandis

**Affiliations:** 1 Department of Laboratory Medicine and Pathobiology, University of Toronto, Toronto, Ontario, Canada; 2 Department of Clinical Biochemistry, University Health Network, Toronto, Ontario, Canada; 3 Department of Pathology and Laboratory Medicine, Mount Sinai Hospital, Mount Sinai Hospital, 60 Murray Street, Toronto, ON, M5T 3L9, Canada

**Keywords:** diagnosis, fascin-1, immunohistochemistry, prognosis, tumor markers

## Abstract

Tremendous progress has been made in recent years towards the understanding, prevention and management of malignant disease, yet cancer remains a leading cause of global mortality and morbidity. Current approaches towards combating this disease include prevention, early detection and various treatment modalities. However, even with implementation of novel therapeutic options and preventative measures, most cancers are currently diagnosed at late stages, when treatment therapies are least effective. In a recent study published in *BMC Medicine*, Tan *et al*. performed a systematic review and meta-analysis to show that fascin-1, an actin-bundling protein, is associated with increased risk of mortality and metastasis in various cancer types. Although the study examined the association of fascin-1 with mortality, time-to-disease progression, lymph node metastasis and distant metastasis in five major cancer types, the clinical implications of these findings are still unclear and many unanswered questions remain.

Please see related research article here http://www.biomedcentral.com/1741-7015/11/52

## Background

According to current World Health Organization statistics, cancer accounts for approximately 7.6 million deaths per annum [1]. Due in part to the expanding and aging of the world's population, this number is expected to rise to a projected 13.1 million deaths per annum in the next two decades [1]. Because cancer continues to pose a major threat to human health, it is essential to develop novel methods for combating it. Current approaches include prevention, early detection, discovery of new treatment modalities and combinations of the three, with a significant portion of research dedicated to development of new treatments. Even with novel therapeutic options and preventative measures in place, the majority of patients present with locally advanced and/or metastatic disease at diagnosis [2]. Tumor metastasis remains a major cause of cancer mortality [3]. A major unmet need in the field of oncology is the ability of biomarkers to allow for early characterization of carcinomas according to their aggressive potential.

According to data by Tan *et al*. data published in *BMC Medicine *[4], fascin-1 is a novel biomarker for aggressive, metastatic carcinomas. Fascin-1 is an actin-bundling protein absent in most normal epithelia but expressed in many human carcinomas [5]. A number of previous studies have implicated fascin-1 as a candidate biomarker for aggressive carcinomas of a large number of cancer types [5,6]. Immunohistochemical (IHC) studies have shown that fascin-1 protein expression is correlated with poor prognosis [6,7]. The objective of Tan *et al*. was to conduct a systematic review and meta-analysis of studies that used IHC to investigate whether fascin-1 could serve as an early marker for identification of aggressive metastatic potential. A total of 26 IHC studies examining five cancer types (breast, colorectal, esophageal, gastric and lung) were selected after a thorough examination of the literature, following strict inclusion criteria. Outcomes such as mortality, disease progression, lymph node metastasis and distant metastasis were examined. The authors approached this systematic review in a standardized, objective manner by applying a number of statistical analyses to this study. Examination of the methodological quality and heterogeneity of the studies, as well as various sensitivity analyses, was carried out.

### Clinical implications of fascin-1

The results of the study by Tan *et al*. revealed that fascin-1 was associated with an increased risk of mortality for breast, colorectal and esophageal cancers. Furthermore, this protein was associated with an increased risk for disease progression in breast and colorectal cancers, whereas an increased risk of both lymph node positivity and distant metastasis was noted for colorectal and gastric cancers (Table 1). The methodological quality of the studies did not greatly influence the overall hazard ratio estimates.

**Table 1:** Summary of the systematic review and meta-analysis by Tan *et al.*[4]

	Breast	Colorectal	Esophageal	Gastric	Lung
**Mortality**	√	√	√	X	X
**Disease progression**	√	√	N/A	N/A	X
**Lymph node metastasis**	N/A	√	X	√	X
**Distant metastasis**	N/A	√	X	√	N/A

Five major cancer types were examined for their association to four clinical outcomes. √: increased risk association with the cancer type; X: no association; N/A: not examined in the 26 studies included in this meta-analysis.

This study raises a number of important questions regarding fascin-1. First, the pathophysiology of fascin-1 needs to be further elucidated as it is unclear how fascin-1 could be associated with an increased risk of both lymph node positivity and distant metastasis in gastric cancer but have no association with mortality. In addition, it is unclear how in lung carcinoma the authors found no association of fascin-1 with lymph node metastasis when figure four in Tan *et al*. show an increased risk (relative risk: 1.98; confidence interval: 1.15, 3.41). With respect to colorectal cancer, it appears that fascin-1 is associated with an increased risk for all four outcomes examined. These findings suggest that fascin-1 may play a major role in colorectal cancer development and the pathophysiology of this needs to be further investigated. Finally, one of the strengths of this review was that all included studies used the same two antibodies for the IHC. It would be interesting to see if the hazard ratio remains the same if the outcomes from these studies are examined according to the individual antibody used.

The authors raised an excellent point about the heterogeneity within carcinoma types - with the prime example being breast carcinoma. Here, each of the studies included different histological types of breast carcinoma. Further analysis of fascin-1 in subtype-specific breast cancer is needed. In fact, well-designed prospective studies are necessary to fully examine the prognostic impact of fascin-1. Furthermore, a number of previous studies have shown that fascin-1 is expressed in many cancer types including cancers of the biliary duct, bladder, brain, breast, colorectal, kidney, liver, ovary, pancreas and so forth [6]. This raises the question as to whether this is a good biomarker, since it is not specific to any tissue type.

### Conclusions and perspectives

The findings of this systematic review and meta-analysis by Tan *et al*. are clinically relevant and important in the field of cancer biomarkers. A thorough study was conducted and the findings raise a number of important questions regarding the pathophysiology of fascin-1 and how it could be implemented for routine clinical care in patients diagnosed with the major carcinomas examined in this study. Although prognostic markers are needed, the cornerstone to combating cancer now and in the future is by early diagnosis. Early diagnosis could result in the cure of many patients with cancer and in more effective treatments for the rest. As fascin-1 is not a secreted or membrane-bound protein, it is unlikely that it will serve as a serological biomarker for any of the cancer types examined.

Recently, we commented on the failure of many new cancer biomarkers to reach the clinic [8]. We grouped the failures into three categories: fraudulent reports, true discoveries that fail to meet the demands of the clinic; and false discoveries. Clearly, time will show if fascin-1 will fall in the second category, or if it will be one of these rare biomarkers that will find its niche in the clinic.

## Abbreviations

IHC: immunohistochemistry.

## Competing interests

The authors declare that they have no competing interests.

## Authors' contributions

VK and EPD contributed to the conception and design of this manuscript. VK drafted the manuscript and EPD made substantial contributions in revising it. VK and EPD read and approved the final manuscript.

## Authors' information

VK is a Clinical Biochemist at the University Health Network in Toronto and an Assistant Professor at the Faculty of Medicine, University of Toronto. EPD is a Professor and Head of Clinical Biochemistry, Department of Laboratory Medicine and Pathobiology, University of Toronto, University Health Network and Mount Sinai Hospital, Toronto, ON, Canada. Both have an interest in novel biomarker discovery and validation using proteomics and mass spectrometry. Please refer to our website for more information: http://www.acdclab.org.

## Pre-publication history

The pre-publication history for this paper can be accessed here:

http://www.biomedcentral.com/1741-7015/11/53/prepub
